# circRNAs in Endometrial Cancer—A Promising Biomarker: State of the Art

**DOI:** 10.3390/ijms25126387

**Published:** 2024-06-09

**Authors:** Karolina Włodarczyk, Weronika Kuryło, Anna Pawłowska-Łachut, Wiktoria Skiba, Dorota Suszczyk, Paulina Pieniądz, Małgorzata Majewska, Ewa Boniewska-Bernacka, Iwona Wertel

**Affiliations:** 1 Independent Laboratory of Cancer Diagnostics and Immunology, Department of Oncological Gynaecology and Gynaecology, Faculty of Medicine, Medical University of Lublin, Chodźki 1, 20-093 Lublin, Poland; weronikakurylo@student.umlub.pl (W.K.); anna.pawlowska@umlub.pl (A.P.-Ł.); wiktoria.skiba@umlub.pl (W.S.); dorota.suszczyk@umlub.pl (D.S.); paulina.pieniadz@umlub.pl (P.P.); iwona.wertel@umlub.pl (I.W.); 2 Department of Virology and Immunology, Institute of Biology and Biotechnology, Maria Curie-Skłodowska University, Akademicka 19, 20-031 Lublin, Poland; 3 Department of Industrial and Environmental Microbiology, Institute of Biology and Biotechnology, Maria Curie-Skłodowska University, Akademicka 19, 20-031 Lublin, Poland; malgorzata.majewska@mail.umcs.pl; 4 Medical Department, Institute of Medical Sciences, University of Opole, Oleska 48, 45-052 Opole, Poland; boniesia@uni.opole.pl

**Keywords:** circRNA, biogenesis of circRNAs, function of circRNAs, endometrial cancer, biomarker

## Abstract

Endometrial cancer (EC) is one of the most common malignant tumors among women in the 21st century, whose mortality rate is increasing every year. Currently, the diagnosis of EC is possible only after a biopsy. However, it is necessary to find a new biomarker that will help in both the diagnosis and treatment of EC in a non-invasive way. Circular RNAs (circRNAs) are small, covalently closed spherical and stable long non-coding RNAs (lncRNAs) molecules, which are abundant in both body fluids and human tissues and are expressed in various ways. Considering the new molecular classification of EC, many studies have appeared, describing new insights into the functions and mechanisms of circRNAs in EC. In this review article, we focused on the problem of EC and the molecular aspects of its division, as well as the biogenesis, functions, and diagnostic and clinical significance of circRNAs in EC.

## 1. Introduction

Endometrial cancer (EC) is the most common gynecologic cancer and the sixth most common malignancy in women worldwide after breast, lung, colorectum, cervix uteri, and thyroid cancers. According to the World Health Organization (WHO), in 2022, as many as 420,368 women were diagnosed with endometrial cancer, and unfortunately, this malignancy caused 97,723 deaths [1]. The highest cancer rates are observed in North America (73,977 new cases) and Europe (124,874 new cases), where the age-standardized rate (ASR) is 17.6 per 100,000 females and 29.7 per 100,000 females, respectively [2,3,4]. The number of new cases of EC increases every year, and the forecasts regarding their number are very disturbing. According to the International Agency for Research on Cancer (IARC), it is estimated that by 2045 the number of new cases of EC will increase by 65% worldwide compared with the number of cases recorded in 2022 [4]. Over the past few decades, the incidence of EC has increased by over 100% [5,6]. Disturbing data about the constantly increasing number of new cases and deaths indicate that EC has become an extremely important problem for medicine around the world. Terrifying incidence and mortality data, as well as disturbing forecasts regarding EC, force the need to search for newer diagnostic methods and biomarkers that will accelerate the diagnosis and prognosis of patients.

The first symptoms of EC that women report are vaginal bleeding after menopause or, in women who are still menstruating, abnormal bleeding between periods [7]. EC is diagnosed by performing a histopathological examination of the material taken during a biopsy. However, collecting material for testing involves patients, among others, with pain or discomfort, and the treatments may lead to various complications (e.g., uterine perforation). Current EC diagnostics are invasive, painful, uncomfortable, and may be dangerous for women who have not yet given birth. Therefore, it is necessary to find a new, non-invasive diagnostic tool that will bring greater benefits to patients and clinicians and, above all, enable a quick and easy diagnosis of EC [7,8].

Non-coding RNAs (ncRNAs) are transcripts or their elements that control many biological processes in the cell. ncRNAs cannot have the ability to encode proteins because they are not subject to post-transcriptional modifications of mRNA [8,9,10,11]. One group of ncRNAs is circular RNAs (circRNAs), belonging to the endogenous class. circRNAs were first discovered in the 1970s and were considered as a splicing error. The use of high-throughput sequencing techniques and bioinformatics methods enabled the identification of circRNAs in various human tissues and body fluids. circRNAs have many important functions, including controlling the amount of miRNA in cells, as well as performing regulatory functions by taking part in post-transcriptional processes or being involved in protein translation [9,10]. They differ from other types of RNAs in their covalent structure, a closed loop generated from mRNA in the process of reverse splicing [10,11]. This special type of splicing enables the covalent connection of the 5′ end (exon/intron) with the 3′ end [8,9,12,13,14,15]. circRNAs are molecules that are ubiquitous in eukaryotic cells that are characterized by high resistance to enzymatic degradation by RNase R. The high stability of these molecules comes from the lack of free ends specific to linear RNAs [14,16,17]. circRNAs may be aberrantly expressed, as is the case in malignant tumors. It is now known that these molecules are associated with numerous processes, such as proliferation, migration, invasiveness, and drug resistance in many cancers, including EC [17,18]. Due to their properties, circRNAs are a very attractive and interesting diagnostic, therapeutic, and prognostic tool that can be used in many cancers, especially EC.

This work aims to present the latest achievements and information in the field of EC as well as to draw attention to the possibilities of using circRNA molecules in its diagnosis, treatment, and prognosis.

## 2. EC Risk Factors

EC develops as a result of uncontrolled growth of endometrial cells (lining the inside of the uterus). This malignancy mainly affects post-menopausal women, and the average age of diagnosis is around 60 years of age [8,19,20,21]. However, EC can also affect younger, still menstruating women under the age of 45 years; fortunately, this is less common, but the number of new cases affecting this group of women has doubled. Currently, more and more cases of EC are observed in women under 50 years of age (approx. 85%), and even under 40 years of age as well (approx. 5%) [5,6]. The main reasons for the increasing incidence of endometrial cancer in highly developed countries are the high percentage of people suffering from obesity, lack of physical activity, increasing stress, environmental pollution, and extended life expectancy [8,14,22]. Factors that strongly increase the likelihood of developing EC are primarily age and obesity. However, the risk of EC is also associated with factors such as metabolic syndrome, race, exposure to estrogens, reproductive factors, genetic predisposition, tamoxifen therapy, and lifestyle [14,19,23,24].

### 2.1. Obesity

Obesity is the most important and best known risk factor for malignant EC (risk ratio 1.52). In developing countries, there is a noticeable correlation between increasing body mass index (BMI) and increasingly common EC [5,25]. Both being overweight (BMI between 25 and 29.9) and having obesity (BMI < 30) increase the risk of EC by approximately 1.4% and 3.3%, respectively. Additionally, each five-unit increase in BMI in adulthood increases the risk of EC by approximately 80% [26,27]. The underlying mechanism of the increased risk of EC due to obesity is the excessive conversion of androgens to estrogens in adipocytes, which stimulates the proliferation of endometrial cells, endometrial hyperplasia, and carcinogenesis [28]. However, on the other hand, there is a correlation when the process of weight loss in obese women is performed, where their risk of EC is reduced [27]. Women with a high BMI are at increased risk of chronic anovulation, resulting in excess exposure to estrogen [22]. In obese people, adipokines (including leptin, TNF-a, IL-6, IL-8) are produced, and oxidative stress and a pro-inflammatory state persist, which promote carcinogenesis and an increased risk of EC [29,30].

### 2.2. Metabolic Syndrome

Metabolic syndrome (MS) is the coexistence of various factors that increase the risk of developing atherosclerotic cardiovascular diseases and type 2 diabetes. MS risk factors include hypertension, obesity, lipid disorders, prediabetes, and diabetes. Diabetes and obesity-related hypertension increase the risk of EC. Moreover, insulin resistance and hyperglycemia lead to abnormalities in insulin-like growth factor (IGF-1) signaling and activation of the mammalian target of rapamycin (mTOR) through the pro-oncogenic PI3K/AKT/mTOR pathway, leading to the development of cancer [22]. Additionally, MS diseases are characterized by almost twice the incidence of EC [31,32,33].

### 2.3. Race

In the United States, the lifetime risk of developing EC in white women is almost 60% higher than in African-American women (2.88% and 1.69%, respectively) [20,34]. Type II EC is less common in white women than in black women [35]. Wilhite et al. [36] showed that black patients with endometroid EC show less frequent mutations in the ARID1A, PTEN, or PIK3CA genes, making the black race have a potentially high risk of EC [36]. In turn, Pearce et al. [37], conducting a pilot study, concluded that in black women with endometrioid EC grades 2 and 3, they had individual differences at the level of pathways (e.g., activated opioid signaling, inhibited corticotropin-releasing hormone signaling) and genes compared with white women. The researchers also noted that regardless of BMI, there were many differences between races [37]. However, further research in this direction is necessary.

### 2.4. Estrogen Exposure

Excessive exposure to estrogens, both endogenous (e.g., in the case of chronic anovulation) and exogenous (e.g., hormone replacement therapy), increases the risk of hormone-dependent EC. This group includes selective estrogen receptor modulators, i.e., tamoxifen, which reduces the risk of breast cancer [19,25]. Additionally, estradiol is involved in carcinogenesis by activating IGF-1 and epidermal growth factor (EGFR) receptors. As a consequence, AKT kinase is activated in the PI3K/AKT/mTOR pathway, which causes the cancerous transformation of endometrial cells [26,38].

### 2.5. Reproductive Factors

Nulliparity, older age at last birth, early menarche, and late menopause contribute to the prolongation of exposure to endogenous estrogens, increasing the risk of EC in these women. However, breastfeeding and taking oral contraceptives contribute to reducing the risk of developing EC [20,23,27]. Polycystic ovary syndrome (PCOS) increases the risk of EC in women by 2.7 times. Additionally, women diagnosed with PCOS show altered miRNA expression, including an increased expression of miR-27a-5p in serum, which is an important factor in the migration and invasiveness of EC cells through the Smad4 protein [23,27].

### 2.6. circRNA and EC’s Risk Factors

There are limited studies regarding the role of circRNAs in the pathogenesis of EC. Study by Takenaka et al. [39] indicate that the circRNA expression profile in EC tissues of obese patients is changed and is characterized by a 40% lower number of circRNAs compared with tissues not changed by cancer. Ye et al. [40] showed that the expression of circRNAs in grade 3 ECs differs significantly compared with healthy tissues. It was found that hotspot genes responsible for circRNA transcription can cause changes in circRNA expression between EC and healthy tissue [39,40].

However, it can be assumed that circRNA molecules play an important role in the pathophysiology of EC, among others, through molecular mechanisms regulating gene expression or the functioning of cancer cells, e.g., the sponge mechanism for miRNAs and regulation of the PI3K/AKT/mTOR signaling pathway [8,12,18,41]. Despite many studies that indicate the role of circRNAs in the development and progression of cancer, their role in the pathogenesis of EC is unknown and is a promising area of research worthy of interest.

## 3. Genetics Background of EC

Inherited genetic syndromes, such as the more common Lynch syndrome (LS) and Cowden syndrome (CS), are associated with the risk of EC [42,43]. Hereditary non-polyposis colorectal cancer (LS) is an autosomal-dominant disease characterized by germline mutations in the MMR genes (MLH1, MSH2, MSH6, PMS2) or hypermethylation of the MLH1 promoter [24,26,42,44,45,46]. LS is associated with an increased risk of colorectal cancer (CRC) and EC as well as, among others, stomach or small intestine cancers [47,48]. In the case of germline mutations, including in the MLH1 and MSH2 genes, the risk of EC increases by 40% to 60% throughout life. The frequency of mutations in the MSH2, MLH1, and MSH6 genes among LS ECs is 50–60%, 24–40%, and 10–13%, respectively [29,46,49].

CS is an autosomal-dominant genetic syndrome caused by mutations in the PTEN anti-oncogene. CS increases the lifetime risk of EC by 19–28% [20]. CS is characterized by the risk of, among others, thyroid cancer and breast cancer. It is observed that the occurrence of somatic mutations in the PTEN gene is more often reported in spontaneous cases of EC than PTEN mutations arising in the germline, which are less common [42].

## 4. Heterogeneity of EC

Endometrial cancer is a heterogeneous tumor. Historically, in 1983, Bokhman distinguished two subtypes of EC—type I and type II [22,29,35]. The first type, estrogen-dependent endometrioid adenocarcinoma, is the most common subtype (accounts for 80 to 90% of all diagnoses) and has no genetic determinants. This type is characterized by a better treatment prognosis, with a 5-year survival rate of about 90%. Risk factors that increase type I of EC include hyperestrogenism, obesity, metabolic syndromes (diabetes, hypertension), infertility due to PCOS, or nulliparity [19,35,50].

Type II is a non-estrogen-dependent, non-endometrioid adenocarcinoma [50,51]. There are different histological types of this EC, including serous carcinoma, clear cell carcinoma, undifferentiated tumors, and mixed tumors [49,51]. Type II is characterized by high mortality and, unlike the first type, has an unfavorable prognosis, where the 5-year survival rate is approximately 50%. It is most often diagnosed in older and non-obese women [20]. However, despite the ease of classification, such a division is not entirely ideal in the case of such a heterogeneous group of cancers as EC [35,52].

## 5. The Strengths and Weaknesses of the FIGO Classification of EC

The first FIGO (International Federation of Gynecology and Obstetrics) endometrial cancer staging system was published in 2009. Since then, knowledge about EC has expanded with much important information that contributes to a better understanding of the pathology and molecular aspects of EC [22,53,54]. The update of the EC FIGO 2023 classification system is still based on the anatomical aspect of the disease in the affected organ; however, it introduces new, non-anatomical parameters for assessing the stage of cancer (including stage I and II) [53,54,55]. It is also extremely important to add a division into categories III and IV, which take into account the location and size of the cancer. Due to a lot of new information that has appeared since the last publication in 2009, FIGO 2023 has introduced another key change, which is the division of patients into categories with a good prognosis or patients with features of cancer predicting a worse prognosis [53]. FIGO 2023 presents both the strengths and weaknesses of its update. One of its great advantages is, among others, the expansion of the categorization of diseases in stages II, III, and IV, making it possible to take into account different types of EC spread, which is extremely important from a therapeutic point of view. The new classification system did not eliminate its weaknesses. According to McCluggage et al. [53], the new system is more complicated than its previous version, which may lead to many difficulties, including in attempts to compare previously diagnosed and currently ill patients in clinical or epidemiological terms. Due to new reports that have emerged since the last EC classification and staging system was published, a new endometrial cancer classification system was published in June 2023. FIGO 2023 was presented as an update and modification of the previous system, which places a strong emphasis on reflecting current findings and reports [55]. The new FIGO classification is an attempt to respond to the latest news that has emerged since the last classification system was published. It allows EC staging to become more accurate and personalized, more precisely tailoring treatment therapies [53,54,55].

## 6. Molecular Subtypes of EC

The results of a comprehensive genomic and proteomic analysis of endometrial cancers conducted by The Cancer Genome Atlas (TCGA), published in 2013, proposed a new division of EC, different from the previous Bokhman classification [52,55,56]. This integrated analysis provided deeper insight into the biological molecular nature of ECs. The analysis identified four new categories: (1) ultramutated *POLE*, (2) hypermutation/high microsatellite instability, (3) high copy number, and (4) low copy number of somatic alterations [52,53,55,56].

The molecular EC classification approach proposed by TCGA is very valuable in prognosis [55,56]. The detection of the *POLE* mutation means a favorable prognosis, regardless of the tumor grade. DNA mismatch repair (MMRd) and no specific molecular profile (NSMP) mutations indicate an intermediate prognosis, while p53abn mutations have the worst prognosis. This analysis provides a lot of necessary information about EC, which may directly influence the therapeutic processes of patients.

### 6.1. POLE-Mutated Subtype of EC

The catalytic subunit of epsilon DNA polymerase is catalyzed by the *POLE* gene, which is the central catalytic subunit of epsilon DNA polymerase. During DNA replication, DNA polymerase-ε and polymerase-δ are responsible for the synthesis of both strands (leading strand and lagging strand) [57,58]. It is believed that polymerase-ε is involved in the synthesis of the leading strand and also plays a key role in the repair and correction of newly synthesized DNA strands. Mutations occurring in the exonuclease domain of epsilon polymerase lead to an impaired 3′ to 5′ correction function [59]. This results in a loss of replication fidelity and a high mutation frequency and consequently leads to genome instability. The most dangerous variants of the *POLE* gene were detected in exons 9, 13, 14, and 32, which may differently affect the activity of 3′ to 5′ exonucleases. Mutations in the *POLE* gene occur in approximately 7–9% of all endometrial cancers and are characterized by an almost 100-fold increased mutational load [22,29,58,60]. Tumors with *POLE* mutations are characterized by internal morphological heterogeneity and exhibit heterogeneous morphology. Above all, they affect younger, slimmer women with a normal BMI. Despite often having the characteristics of high-risk tumors, they have a more favorable prognosis, which may be the result of an ultramutated phenotype and sensitivity to adjuvant therapy [58].

### 6.2. Mismatch Repair-Deficient Subtype of EC

Microsatellite sequences: Microsatellites are short-tandem repeats occurring in both coding and non-coding regions of the genome. Microsatellites play a promoter role in the genome during the DNA replication process [61]. A large accumulation of errors in satellite sequences results in defects in replication fidelity and a malfunctioning post-replication DNA repair system, consequently leading to microsatellite instability (MSI) [51,57,58]. The emerging defects are the result of mutations in mutator genes (dMMR), responsible for maintaining DNA integrity. More than 30% of EC cases are dMMR/MSI-H endometrial cancers, which are characterized by the loss or abnormal expression of MMR protein [62]. dMMR/MSI-H tumors have an intermediate prognosis and higher TMB16. Lymphatic vascular space infiltration (LVSI) is also more frequently observed. This group of ECs is not associated with high BMI but may occur in a very wide age range. In patients with Lynch syndrome, EC appears earlier than in sporadic cases [22,57,63].

### 6.3. Non-Specific Molecular Profile Subtype of EC

EC without *POLE*, dMMR, and *TP53* mutations are diagnosed as an NSMP, also called a low copy number (CNL) [22,57]. NSMP occurs in 50% of EC cases and has a worse prognosis than *POLE*- and dMMR-mutated EC types [58]. This subtype is characterized by low somatic mutation burden and low copy number changes, and they histologically demonstrate a low degree of malignancy and an intermediate prognosis [22,51,58]. Additionally, it is accompanied by a high expression of estrogen receptors (ERs) and progesterone receptors (PRs). Patients diagnosed with this EC subtype have the highest BMI [51]. EC with the NSMP subtype is associated with *CTNNB1* mutation (gene encoding beta 1 catenin), which is associated with more distant recurrences in PORTEC cohorts. It was found that NSMP is associated with a mutation of the L1 cell adhesion molecule (L1CAM), which is a glycoprotein involved in the migration of cancer cells [57]. According to the Proactive Molecular Risk Classifier for Endometrial Cancer (ProMisE), this molecule is a key indicator of EC with unfavorable survival. Patients with NSMP, L1CAM-positive have a comparable risk of death as in the case of the p53 mutation subtype. The NSMP group is also associated with mutations in the PI3K/Akt/mTOR signaling pathway, which is associated with ER+ and PR+ [22,57,58,64].

### 6.4. P53 Abnormal Subtype of EC

This EC subpopulation is characterized by high somatic copy number changes and is correlated with a high degree of tumor malignancy as well as the occurrence of mutations in the *TP53* gene, encoding the p53 protein [65,66]. Commonly, this protein is considered the guardian of the human genome; it is responsible for the stability of the genetic material through, among others, the correct transcription of many genes and regulation of cell cycle control points, DNA repair, and apoptosis. The appearance of mutations in the *TP53* gene results in the p53 protein having oncogenic functions, promoting cancer proliferation or resistance to treatment. EC with a *TP53* mutation shows many molecular similarities to cancers, such as high-copy fallopian tube cancers, serous fallopian tube cancers, and basal-like breast cancers. Unlike the above-mentioned cancers, EC with p53 abn mutation is characterized by recurrent mutations in the *PIK3CA*, *PPP2R1A*, and *FBXW7* genes and a reduced probability of mutations in the *BRCA1* or *BRCA2* genes [51]. This molecular subtype includes serous carcinomas, sarcomatoid carcinomas, clear cell carcinomas, grade 3 carcinomas, and grade 1–2 carcinomas. EC p53 abn is associated with older age and lower BMI. Although this subtype is detected in approximately 15% of all EC, it correlates with an advanced stage and worse prognosis and is also responsible for 50–70% of EC deaths. At the same time, it should be kept in mind that EC is a highly heterogeneous group in which a mutation in the *TP53* gene may be a later event during tumor progression in dMMR- or *POLE*-mutated tumors. In such a situation, the cancer is classified into an appropriate group, either dMMR- or *POLE*-mut. According to the results obtained from the PORTEC-3 trial, patients who received chemotherapy in addition to radiotherapy had a better response to treatment [51,57,66].

## 7. Biogenesis of circRNAs

**Figure 1:**
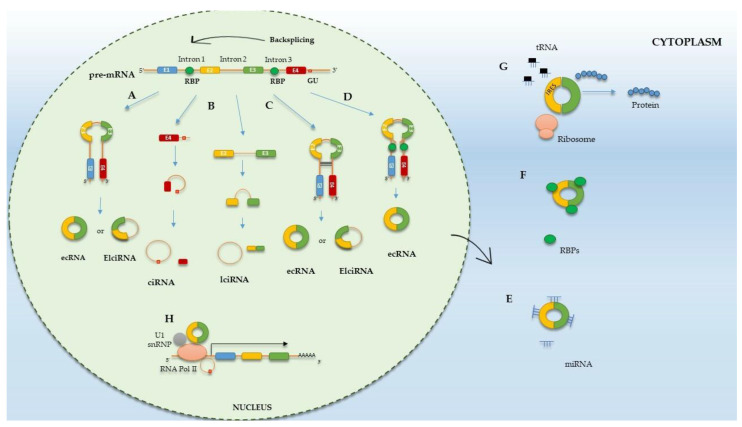
Biogenesis and functions of circular RNA. The ways of forming circRNAs: (A) Lariat-driven circularization. (B) Intronic lariats. (C) Intron pairing-driven circularization. (D) RBP mediated circularization. The functions of circRNAs: (E) circRNAs can regulate mRNA expression by acting as miRNA sponges. (F) circRNAs can act as protein sponges. (G) Some circRNAs have the ability to translate into protein. (H) circRNAs can interact with RNA Pol II to enhance and regulate parental gene transcription.

In the process of mRNA maturation in eukaryotic cells, post-transcriptional removal of introns and splicing of exons from the precursor mRNA (pre-mRNA) occurs in the presence of a multi-protein complex—the spliceosome [12,67]. The mature mRNA created in this way can participate in the translation process. circRNAs are also formed from pre-mRNA in the presence of a spliceosome in the cell nucleus but through reverse splicing [8,68]. The basis of this process is the exchange of the junction between the exon splice donor site and the upstream exon acceptor site. In such a case, it is possible to create a single- or multi-exon circular circRNAs molecule, characterized by the lack of a cap at the 5′ end and a poly(A)-tail at the 3′ end, in which the ends are covalently connected to each other, thus creating a back splicing [10,12,67]. The regulatory mechanisms of splicing in the formation of circRNAs are different from linear isoforms [8,10,12]. circRNA molecules are characterized by a significant diversity of reverse splicing events, which are catalyzed by the typical spliceosome mechanism in different cell lines [8,12]. Depending on the splicing method, circRNAs can be categorized into four types: exonic circRNAs (ecircRNAs), exon-intronic circRNAs (ElciRNAs), intronic circRNAs (ciRNAs), and intergenic circRNAs (lciRNAs) (Figure 1A–D) [8,9,12,18,41]. circRNA molecules are highly stable and expressed in both intra- and extra-cellular fluids [8]. The expression of circRNAs is kept at a low level. However, in most cases, an increase in the expression of circRNAs is observed in the development of EC. So far, six mechanisms of circRNAs biogenesis have been proposed: the exon skipping mechanism (also called lariat-driven circulation), non-canonical splicing mechanism (intron pairing), interaction mechanism via RNA-binding proteins (RBPs), circulation based on splicing of transported RNA (tRNA), direct circulation of lariat introns, and a mechanism driven by ribosomal RNA (rRNA) splicing [10,12,67,68]. However, there are currently hypothetical models of the mechanisms of circRNA biogenesis have been accepted and will be described in this article.

The first model of biogenesis is a mechanism based on exon skipping during pre-mRNA transcription, also called lariat-driven circulation [9,69]. In this model, reverse splicing results in the skipping of one or more exons in the mature mRNA. In this mechanism, two non-adjacent exons are joined to form a lariat structure, and its structure promotes circulation and the production of intronic circRNAs (ciRNAs) [12,13,68].

The second model of circRNA biogenesis is circulation driven by intron pairing. In the mechanism, two introns flanking pre-mRNA exons contain inverted complementary sequences capable of pairing, leading to the production of various circRNAs (e.g., ecircRNAs, ElciRNAs) [12,67,68]. Furthermore, the longer the introns in the circRNA flanking sequences, the more that ALU repeat elements assist in circRNA formation [10,12,68].

Another mechanism of circRNA formation is interaction with RBPs. This model is based on protein factors capable of binding to pre-mRNA, connecting flanking introns through protein dimerization, resulting in the formation of an RNA loop [10,12,70]. The most popular RBP is slicing regulator protein 1 (MBNL1), which can attach to conserved MBL binding sites and connect flanked introns through dimerization, accelerating circRNA circulation. Other RBP proteins also act similarly, i.e., nuclear factor 90 (NF90) and nuclear factor 110 (NF110) and adenosine deaminase 1 acting on RNA (ADAR1), promoting the process of reverse splicing [10,12,67].

## 8. Function of circRNAs

circRNA molecules may play a multifaceted role in the pathogenesis of EC thanks to a wide range of functions and biological mechanisms. The main mechanism is the action of circRNAs as a sponge for miRNAs, binding to them and thus preventing the miRNA from attaching to a particular mRNA. Such a mechanism leads to the release of target genes from miRNA-mediated repression as well as post-transcriptional repression [10,14,41,71]. An example of this effect of circRNAs is research by, among others, Zhou et al. [72], who revealed that circRNA hsa_circ_0039569, through the sponging of miR-197, indirectly regulates the expression of the HMGA1 gene, promoting the proliferation, migration, and invasion of EC cells. The results of this study point to a new therapeutic target for EC. Additionally, Shen et al. [73] showed that hsa_circ_0002577 is associated with poor patient prognosis, high FIGO III/IV stage, and metastases. hsa_circ_0002577 may be a sponge for miR-197, regulating the expression of the CTNND1 gene, which promotes the proliferation, migration, and invasion of endometrial cancer cells through the hsa_circ_0002577/miR-197/CTNND1/Wnt/β-catenin signaling axis, which may be a potential therapeutic target [73].

The vast majority of circRNAs located in the cytoplasm regulate gene expression through miRNA sponging. However, another mechanism for the regulation of gene expression by circRNAs has been discovered. A small part of these molecules may remain in the cell nucleus, regulating gene expression at the transcriptional level [12,14,67]. This mechanism is used by circRNAs of exonic, intronic, and exon-intronic origin. As indicated by the research of Li et al. [74], circCEIF3J and circPAIP2 (EIciRNA), through their presence in the cell nucleus, promote the expression of parental genes through interactions with small nuclear ribonucleoprotein U1 (snRNP). The formed EIciRNA-U1 snRP complex interacts with RNA polymerase II to regulate gene transcription [74].

circRNAs interact with RNA-binding proteins (RBPs), influencing their functioning and various cellular processes [12,14]. Zhang et al. [75] showed an increased expression of circRAPGEF5 in EC tissues compared with healthy tissues. They also showed that circRAPGEF5 promoted rapid EC cell proliferation and interacted with the C-terminal domain of the RNA-binding protein fox-1 homolog 2 (RBFOX2). This results in difficult binding of RBFOX2 to pre-mRNA and, consequently, leads to the resistance of EC cells to ferroptosis, which may be an interesting therapeutic target [75]. Recent research by Shi et al. [76] identified a new circRNA with an N6-methyladenosine (m6A) methylation modification, hsa_circ_0084582 (circCHD7). Scientists determined that circCHD7 is upregulated in EC tissues and interacts with the insulin-like growth factor RNA-binding protein 2 (IGFBP2), resulting in increased expression of platelet-derived growth factor beta (PDGFRB) receptor mRNA. circCHD7, via the circCHD7/IGF2BP2/PDGFRB axis, activates the JAK/STAT signaling pathway, consequently leading to the promotion of EC cell proliferation. This mechanism could become a promising therapeutic target in the treatment of EC [76].

Although circRNAs belong to the group of non-coding RNAs, it has only recently been known that they can actively participate in the protein translation process [10,41,67]. Despite the lack of a cap at the 5′ end and a poly(A)-tail at the 3′ end, they are equipped with an internal ribosome entry site (IRES), which suggests their potential role in translation [41]. One of the few circRNAs that plays a role in myoblast proliferation is circ-ZNF609 [77]. As shown by Legnini et al. [77], circ-ZNF609 has a 753 nt open reading frame (ORF) that can be translated in a cap-independent but splicing-dependent manner. Another example of circRNA molecules that can be translated is circβ-catenin. The result of translation is that protein affects HCC cell growth by activating the Wnt pathway [78]. Both peptides and proteins produced in circRNA translation influence cancer development, but at present, little is known due to the limitations of research methods. Therefore, further research in this direction is necessary.

The modes of schematic action of circRNAs are presented in Figure 1E–H.

## 9. The circRNAs in Immunotherapy

Immunotherapies have revolutionized cancer treatment and have become one of the fastest-growing cancer therapies over the past decades. As studies have shown, circRNAs may play an important role in various cancer immunotherapeutic methods, including cancer vaccines, immune checkpoint inhibitors (ICIs), or chimeric antigen receptor (CAR) [79].

Cancer vaccines based on circRNA have many advantages over miRNA-based vaccines because of the covalently closed structure of circRNA and the possibility of undergoing the translation process, which significantly improves their stability [69,79]. Additionally, circRNA-based vaccines do not enter the cell nucleus, unlike mRNA, which can be transcribed and may increase the risk for patients when using them. circRNAs can be used in vaccines as adjuvants or antigens, which improves the effectiveness of immunotherapy [79,80,81].

The use of ICIs include but are not limited to anti-PD-1, anti-PD-L1 agents, are currently among the most effective approved immunotherapies used in cancer treatment. As indicated by Yu et al. [79], circRNA molecules can regulate the expression of immune checkpoints (ICPs) in association with the response to ICIs treatment. First, circRNAs can increase the expression of ICPs, including PD-1/PD-L1 or TIM3, which in turn causes increased resistance to ICIs [79,82,83]. Secondly, they can increase the expression of other molecules, e.g., PKP3, which moderates the tumor immune response [84].

circRNA molecules contribute to the increased effectiveness of CAR-based immunotherapy. It has been shown that circRNAs influence the antitumor activity of NK cells, among others, by increasing the regulation of NK’s ligand expression on tumor cells and reducing cytokine secretion [79,85,86]. Additionally, circRNAs can promote the polarization of macrophages to M2 as well as reduce their secretory functions [75,79,86]. However, the aspect of the involvement of circRNAs in immunotherapy requires further research.

## 10. The circRNAs in EC

Endometrial cancer is one of the malignancies whose mortality is increasing, and IARC forecasts only confirm this. EC has become a very serious problem and challenge for medicine. Therefore, new, effective diagnostic, therapeutic, and prognostic methods are urgently sought. circRNA is characterized by numerous features, such as a covalently closed structure, greater resistance to enzymatic degradation, consisting mostly of exons, or being expressed according to precisely defined patterns specific to cells, tissues, etc. All of these features suggest that circRNAs may play an important role in many processes in cancer cells. Thanks to this, circRNAs may prove to be a very good diagnostic, therapeutic, and prognostic biomarker for EC [12,67,87,88].

Year by year, the number of publications and research on the role of circRNAs in EC increases, and thus, the number of newly discovered circRNA molecules present in cells, tissues, and serum increases. Research by Ye et al. [40] showed that over 75,000 circRNAs are significantly changed in EC tissues compared with the control group. Additionally, scientists showed that the hsa_circ_0039569 and hsa_circ_0001610 molecules were characterized by reduced levels of expression in lower stages of EC than in stage 3. At the same time, scientists found that both molecules were correlated with EC cell differentiation [40]. hsa_circ_0039569 plays an important role in the diagnosis and treatment of EC in three stages [40].

Liu et al. [89] showed that hsa_circ_0011324 has high expression in EC. They also suggest that hsa_circ_0011324 participates in EC promotion, migration, and invasion through the mTOR-targeting miR-497 sponging mechanism, indicating that it may be a diagnostic and therapeutic agent for EC [89]. Shen et al. [73] showed a high expression of hsa_circ_0002577 in EC tissues, which was associated with an advanced FIGO stage, lymph node metastasis, and low patient survival rate. Additionally, researchers demonstrated that hsa_circ_0002577 promotes EC cell proliferation and invasion through the miR-197/CTNND1/Wnt/β-catenin pathway. In conclusion, hsa_circ_0002577 shows potential as a prognostic and therapeutic marker of EC [73]. However, it was found that circRNAs may be associated with the resistance of EC cells to chemotherapy treatment. Yuan et al. [90] showed that hsa_circ_0001860 in EC cells is significantly downregulated, which promotes the development of medroxyprogesterone acetate (MPA) resistance, and showed a negative correlation with the grade of EC and lymph node metastasis. Studies have shown that hsa_circ_0001860 promotes Smad7 expression by sponging miR-520h. Therefore, hsa_circ_0001860 may be a target in the fight against EC resistance to MPA [90]. Another study by Sun et al. [91] showed that circ_0005667 is upregulated in EC cells and increases the level of IGF2BP1 through the miR-145-5p sponging mechanism, which leads to the resistance of EC cells to cisplatin (DDP) treatment. It has been proven that reducing the expression of circ_0005667 increases the sensitivity of EC cells to DDP and inhibits the proliferation, migration, and invasion of cancer cells. Therefore, circ_0005667 may be used as a therapeutic target in chemotherapy treatment [91].

Liquid biopsy is a method that uses human body fluid (e.g., blood, urine) as a sample source for diagnostic tests or monitoring the course of the patient’s disease [92,93,94]. A huge advantage of liquid biopsy is the ability to perform it in real time, and it is a non-invasive method compared with tissue biopsy. It is widely used in the treatment process of patients suffering from, among others, oncological diseases [92]. circRNAs are characterized by appropriate properties (including being covalently closed, also being found in body fluids, and having high stability and high specificity) and constitute an excellent example of a biomarker used in liquid biopsy [92,95]. Xu et al. [96] conducted research on the use of serum circRNAs as EC biomarkers, using serum collected from patients with grade 3 EC. The study identified over 200 circRNAs with different expression, but only hsa_circ_0109046 and hsa_circ_0002577 achieved a 2-fold higher change in expression. The researchers managed to confirm this using qRT-PCR; therefore, circRNAs present in the serum can be used as diagnostic biomarkers for EC [92,96].

The possibility of using circRNAs as biomarkers in EC in practice is still a long way off. Currently, several clinical trials in the field of circRNAs are registered in the NCI clinical trials, including NCT04464122 and NCT05771337, as of the publication date of this study.

circRNAs that have so far been investigated in EC and that are suggested to have diagnostic, therapeutic, or prognostic biomarker potential are presented in Table 1. Due to the fact that several circRNAs can be generated from one mRNA precursor, the table also includes the numeric circBASE ID.

## 11. Conclusions

circRNAs have numerous advantages. In addition to biological functions, they also have economic benefits. The use of circRNAs as biomarkers for routine monitoring of recurrence or progression in patients diagnosed with cancer could effectively reduce healthcare costs. circRNAs, being highly sensitive and highly specific, can reduce the costs of early-stage cancer diagnosis. An additional advantage is the speed of distinguishing low-risk from high-risk patients [12,87,111]. However, it should be noted that there are limitations to the use of circRNAs. This is because the vast majority of known circRNAs are characterized by dysregulated levels in various types of cancer [9,111]. Additionally, the studies were carried out on a small scale, and there is no systematic nomenclature of these molecules. Therefore, further research is necessary to better understand the mechanism of action of circRNA molecules in EC. Further research will enable the development of new diagnostic, therapeutic, and prognostic tools in the form of circRNAs that are effective in the fight against the deadly player, EC.

## Figures and Tables

**Table 1:** The circRNAs in endometrial cancer (EC).

circRNAs	Expression	Binding	Function in EC	Clinical Sample	Type of Study	Experimental Model	Clinical Value	PMID	References
hsa_circ_0039569	Up	miR-542	Oncogene; promotes EC cells proliferation, migration, invasion	Tissue	In vitro	-	Diagnostic	31308756	[40]
circ_0039569	Up	miR-1271-5p/PHF6	The paclitaxel resistance	Tissue	In vitro/In vivo	Mouse	Therapeutic target	36136988	[97]
circCCL22 (hsa_circ_0039569)	Up	miR-543/CDC25A axis	Promotes EC cells progression, proliferation, migration, invasion	Tissue	In vitro/In vivo	Mouse	Therapeutic target	37747673	[98]
hsa_circ_0039569	Up	miR-197/HMGA1 axis	Promotes EC cells progression, proliferation, migration, invasion	Tissue	In vitro	-	Therapeutic target	35130798	[72]
circWHSC1	Up	miR-646/NPM1	Promotes EC cells progression, proliferation, migration, invasion	Tissue	In vitro/In vivo	Mouse	Prognostic biomarker/ Therapeutic target	32378344	[99]
circIFT80(hsa_circ_0067835)	Up	miR-324-5p/HMGA1	Promotes EC cells progression, proliferation, migration, invasion	Tissue	In vitro/In vivo	Mouse	Therapeutic target	33169939	[100]
circIFT80	Up	miR-545-3p/FAM98A	Promotes EC cells progression, proliferation, migration, invasion	Tissue	In vitro/In vivo	Mouse	Therapeutic target	34783205	[101]
circSLC6A6	Up	miR-497-5p/PI4KB	Promotes EC cells progression, proliferation, migration, invasion	Tissue	In vitro/In vivo	Mouse	Diagnostic/Therapeutic target	34258297	[102]
circATP2C1 (hsa_circ_0005797)	Up	miR-298/CTNND1	Promotes EC cells progression, proliferation, migration, invasion	Tissue	In vitro/In vivo	Mouse	Therapeutic target	34852711	[103]
circ_POLA2	Up	miR-31	Promotes EC cells progression, proliferation, migration, invasion	Tissue	In vitro	-	Therapeutic target	34539866	[104]
circZNF124	Up	miR-199b-5p/SLC7A5	Promotes EC cells proliferation, leucine uptake, migration, invasion	Tissue	In vitro/In vivo	Mouse	Therapeutic target	34145797	[105]
circ_0109046	Up	miR-105/SOX9/Wnt/β-catenin axis	Promotes EC cells proliferation, aggressiveness	Tissue	In vitro/In vivo	Mouse	Poor prognostic	33220169	[106]
circWDR26 (hsa_circ_0002577)	Up	MiR-625-5P/IGF1R	Promotes EC cells progression, proliferation, migration, invasion	Tissue	In vitro/In vivo	Mouse	Therapeutic target	32847606	[107]
circWDR26	Up	miR212-3p/MSH2	Promotes EC cells progression, proliferation, migration, invasion	Tissue	In vitro/In vivo	Mouse	Therapeutic target	35897048	[108]
hsa_circ_0002577	Up	miR-197/CTNND1/Wnt/β-catenin axis	Advanced FIGO stage, lymph node metastasis, poor overall survival rate, promotes EC cells proliferation and invasion	Tissue	In vitro/In vivo	Mouse	Poor prognostic/Therapeutic target	31081718	[73]
circTNFRSF21 (hsa_circ_0001610)	Up	miR-139-5p	Promotes radiation resistance of EC cells	Tissue	In vitro/In vivo	Mouse	Therapeutic target	34462422	[109]
circTNFRSF21 (hsa_circ_0001610)	Up	miR-1227-MAPK13/ATF2	Promotes EC cells apoptosis, inhibits cells proliferation	Tissue	In vitro/In vivo	Mouse	Therapeutic target	32299063	[110]
hsa_circ_0001610	Up	miR-646/STAT axis	Promotes EC cells progression, proliferation, migration, invasion	Tissue	In vitro/In vivo	Mouse	Therapeutic target	33822442	[111]
circZNF700 (hsa_circ_0109046)	Up	miR-136/HMGA2	Promotes EC cells proliferation, migration, invasion, EMT	Tissue	In vitro/In vivo	Mouse	Therapeutic target	33173333	[112]
hsa_circ_0000043	Up	miR-1271-5p/CTNND1	Promotes EC cells proliferation, migration, invasion, reduces apoptosis	Tissue	In vitro/In vivo	Mouse	Therapeutic target	33128584	[113]
circ_PUM1	Up	miR-136/NOTCH3	Promotes EC cells progression, proliferation, migration, invasion	Tissue	In vitro/In vivo	Mouse	Therapeutic target	32073729	[114]
circFAT1	Up	miR-21	Increases of EC cells stemness	Tissue	In vitro	-	Therapeutic target	34314629	[115]
circESRP1	Up	miR-874-3p/CPEB4 axis	Promotes EC cells proliferation, migration, invasion	Tissue	In vitro/In vivo	Mouse	Therapeutic target	35317822	[116]
circWEE1 (hsa_circ_003390)	Up	miR-195-5p	Oncogene promotes EC cells proliferation, migration, invasion	Tissue	In vitro/In vivo	Mouse	Therapeutic target	35546509	[117]
circ_0002577	Up	miR-126-5p/MACC1	Promotes EC cells proliferation, migration, invasion	Tissue	In vitro/In vivo	Mouse	Therapeutic target	35103833	[107]
circ_0067835	Up	miR-324-5p/HMGA1	Promotes EC cells proliferation, migration, invasion	Tissue	In vitro/In vivo	Mouse	Therapeutic target	33169939	[100]
circRAPGEF5 (hsa_circ_00016181)	Up	RBFOX2	Oncogene, resistance to ferroptosis reduction of labile iron	Tissue	In vitro/In vivo	Mouse	Therapeutic target	36182807	[75]
circSMAD2	Up	miR-1277-5p/MFGE8 axis	Promotes EC cells progression, proliferation, migration, invasion	Tissue	In vitro/In vivo	Mouse	Therapeutic target	36659830	[118]
circ_0005667	Up	miR-145-5p/IGF2BP1	Promotes cisplatin resistance	Tissue	In vitro/In vivo	Mouse	Chemotherapy target treatment	36728962	[91]
circSEPT9	Up	miR-186	Promotes EC cells invasion, migration, methylation miR-186	Tissue	In vitro	-	Therapeutic target	35777791	[119]
hsa_circ_0011324	Up	miR-497/16-5p	Promotes EC cells progression, proliferation, migration, invasion	Tissue	In vitro	-	Diagnostics/Therapeutic target	35259044	[89]
hsa_circ_0023404	Up	miR-217/MAPK1 axis	Promotes EC cells progression, proliferation, migration, invasion	Cell culture	In vitro	-	Biomarker/Therapeutic target	36352482	[120]
circRIMS	Up	miR-505	Promotes EC cells proliferation	Tissue	In vitro	-	Therapeutic target	35695664	[121]
circCHD7	Up	IGF2BP2	Promotes EC cells proliferation, suppresses apoptosis	Tissue	In vitro/In vivo	Mouse	Therapeutic target	38778089	[76]
circCORO1C (hsa_circ_0000437)	Down	CORO1C-47aa	Inhibits angiogenesis, proliferation, migration, and differentiation of EC cells	Tissue	In vitro/In vivo	Mouse	Poor prognostic/Therapeutic target	34534547	[122]
circESYT2 (hsa_circ_0001776)	Down	miR-182/LRIG2 axis	Promotes EC cells progression, proliferation, migration, invasion	Tissue	In vitro/In vivo	Mouse	Therapeutic target	32863771	[123]
circZCCHC7 (hsa_circ_0001860)	Down	miR-520h/Smad7	Promotes EC cells resistance to MPA	Tissue	In vitro/In silico	-	Target to reverse the MPA resistance	34912799	[90]
hsa_circ_079422	Down	miR-136-5p	Potential role in signaling pathways and the biological processes of EC	Tissue	In vitro	-	Poor prognostic	37408092	[124]
circ_0000437	Down	miR-626/CDKN1B	Lymph node metastasis, high TNM	Tissue	In vitro	-	Biomarker/Therapeutic target	35733309	[125]

## Data Availability

No new data were created or analyzed in this study.
